# miRNA-199a-5p/SLC2A1 axis regulates glucose metabolism in non-small cell lung cancer

**DOI:** 10.7150/jca.67990

**Published:** 2022-04-18

**Authors:** Yikun Xu, Binshu Chai, Xianyi Wang, Zong Wu, Zhitao Gu, Xiaomin Liu, Yiqi Zhao, Tangbing Chen, Zhongliang Ma, Qiangling Sun

**Affiliations:** 1 Lab for Noncoding RNA & Cancer, School of Life Sciences, Shanghai University, 200444, China; 2 Department of Thoracic Surgery, Shanghai Chest Hospital, Shanghai Jiao Tong University, Shanghai, 200030, China; 3 Thoracic Cancer institute, Shanghai Chest Hospital, Shanghai Jiao Tong University, Shanghai, 200030, China; 4 Shanghai New Tobacco Product Research Institute, Shanghai, 201315, China

**Keywords:** miR-199a-5p, SLC2A1/GLUT1, NSCLC, GLUTs, non-coding RNAs

## Abstract

Lung cancer is acknowledged as a common cancer with high morbidity and mortality. MicroRNAs (miRNAs), kind of non-coding single-stranded RNA molecules, can be used in cancer clinical treatments. In this research, miR-199a-5p was seen lowly expressed in NSCLC sera samples. miR-199a-5p suppressed the cell proliferation, migration and arrested cell cycle in NSCLC cell lines. The results showed that SLC2A1 (glucose transporter 1, GLUT1) was a direct target of miR-199a-5p. Downregulation of SLC2A1 could not only inhibit cell proliferation, migration and cell cycle, but also promote cell apoptosis. The data suggests that miR-199a-5p can inhibit glucose metabolism in NSCLC by targeting SLC2A1.This study proves that miR-199a-5p / SLC2A1 can play an essential role in the development of NSCLC by targeting SLC2A1. It puts forward a new approach for clinical treatments of NSCLC.

## Introduction

Lung cancer is a kind of respiratory malignancy with the highest morbidity and mortality worldwide, accounting for 13% of all cancers^1^. In China, the incidence and mortality of lung cancer also increased in recent years^2^. The pathogenesis of lung cancer is complex, including a variety of genetic and epigenetic changes, especially the up-regulation of oncogenes and the down-regulation of tumor suppressor genes^3^. With the continuous development of biotechnology, targeted therapy of lung cancer has a broader development prospect because of the multiple targets of lung cancer and the increasingly obvious limitations of traditional therapies such as chemotherapy^4^. It should find new diagnosis markers or targets for lung cancer.

MicroRNAs (miRNAs), composed of 18-22 nucleotides, are identified as endogenous non-coding RNAs that make major efforts in post-transcriptional gene expression. miR-199a-5p can target a variety of target genes in different tumors and play a significant regulatory effect in cancer cells and cardiomyocytes^5^. Li *et al.* found miR-199a-5p as a suppressor of NSCLC via targeting MAP3K11^6^. Shen *et al.* showed that the expression of miR-199a-5p was decreased in hepatocellular carcinoma (HCC)^7^. Glucose homeostasis is an important player in cancer metabolism^8^. However, whether miR-199a regulates glucose metabolism is unclear. Autophagy is required for glucose homeostasis and lung tumor maintenance.

In this study, we hypothesize that miR-199a suppresses lung cancer via regulating glucose metabolism. Our research showed that miR-199a-5p suppresses NSCLC via targeting SLC2A1 which is a member of the glucose transporter family (GLUTs), playing a significant part in maintaining glucose homeostasis.

## Materials and methods

### Tissue samples and cell culture

The lung cancer samples and related normal samples from 41 NSCLC patients were acquired from Shanghai Chest Hospital and ethical assessment passed. The details of selected patients are provided in supplementary table 1. All kinds of NSCLC cell lines, which include A549, H1299, H1975, PC-9, 95-D, HCC827, and human epithelial kidney cell line HEK-293T were acquired from Cell Bank (Shanghai, China). Two main mediums, Dulbecco's modified Eagle's medium and RPMI-1640 medium were used to culture cells and both of them were added 10% FBS and 100 U/ml penicillin. The environment standard for cell culture is 37 °C and 5% CO_2_.

### Cell transfection

Ribobio Company (Guangzhou, China) provides Both miRNA mimics and siRNAs. With Lipofectamine 2000, 50 nM of miR-199a mimic / siSLC2A1 and those related NC were used to transfect cells respectively.

### Cell proliferation assay

Cell Counting Kit-8 (CCK-8) assay kit (Dojindo, Tokyo, Japan) was used to represent cell proliferation. 2 × 10^3^ cells in each well were placed into a 96-well plate and incubated at 37 °C for 3 days. Then CCK-8 solution was added to each well before incubating for 2 h at 37 °C in 5% CO_2_. The plate was measured at 450 nm absorbance once a day for 3 - 4 times.

### Colony formation assay

Cells were counted in a number of 700 and were plated in each well of a 6-well plate. The whole plate was incubated for 2 weeks in an environment of 37 °C and 5 % CO_2_.

Methanol was used to fix the colonies and 0.5 % w/v crystal violet was used to stain. Wells were flushed with water and then colonies were counted.

### Cell migration assay

Cells were counted in 2×10^5^ and then placed in a 6-well dish. A sterile pipette tip was used to scratch a single wound in the middle of each well. The migrations happened and were photographed after 24 h.

### Cell cycle assay

Cells were stored in cold ethanol and were treated with RNase A and 2 × PI for 15 min without light the next day. Analyses of the cell cycle were made by XDP flow cytometer (Beckman Coulter, USA) and FlowJo 7.6.2.

### Cell apoptosis

The cell density of each well in the 12-well plate was about 80%~95% and then it was placed under ultraviolet for 2 h. Before adding 1×Binding Buffer and the dye, PBS was added to each well to wash/clean the well. Cells were incubated for 30 min without light and filtered with 50 μm nylon membrane before apoptosis was detected by flow cytometry.

### Dual luciferase Reporter Assay

The SLC2A1 3'UTR containing the targeted binding of miR-199a-5p was inserted into the firefly luciferase expression vector pGL3 miReport to construct the wild-type recombinant plasmid, denoted as PGL3-SLC2A1-WT-3'UTR. Meanwhile, the miR-199a-5p and SLC2A1 3'UTR binding sites were selected for interval mutation, and the corresponding mutant recombinant plasmid was constructed, denoted as pGL3-SLC2A1-Wut-3'UTR. In order to verify that SLC2A1 was the direct target gene of miR-199a-5p, all plasmids were co-transfected into HEK-293T cells, which were later verified by the Dual-Luciferase Reporter Assay System (Promega) kit.

### Quantitative real-time PCR (qRT-PCR)

TRIzol Regent was used to extract the whole quantity of RNA from cells and PrimeScript^®^ miRNA SuperMix Kit (Transgen, Beijing, China) was used to Reverse transcript miRNAs, followed by QuantiMiR cDNA Kit(TaKaRa, Dalian, China) constructing the entire mRNAs cDNA library. The level of RNA was detected by qRT-PCR with SYBR. The endogenous control of mRNA was 18S while that of miRNA was U6.

### Western blot

RIPA Lysis Regent (MesGenBiotech, Shanghai, China) was used to test the protein which was extracted from the cells and Bradford Assay Kit (BioRad, Hercules, USA) for quantification. SDS- PAGE was used to detect protein which was then transferred into 0.22 μm PVDF membrane. The membrane was divided and incubated in Rabbit anti-SLC2A1 (1:1000) and rabbit anti-GAPDH (1:1000) respectively at 4 °C overnight. On the following day, the membrane was moved into HRP-labeled secondary antibody (1:10,000) and was also incubated for 1.5 h. After washing, chemiluminescence ECL was added to imaging.

### Glucose uptake assay

2-NBDG (Glpgio, US), a kind of fluorescent glucose analog for visualizing glucose uptake into living cells, was used to measure glucose uptake 2 × 10^5^ pre- treated cells were moved into a 24-well plate and incubated overnight. On the next day, cells were starved for 2 h and incubated without glucose and 3 μl 100 nM 2-NBDG at 37 °C for 40 min. The samples were washed three times with PBS. The fluorescence intensity was determined by an automatic enzyme plate analyzer at the excitation wavelength of 485 nm and the reflection wavelength of 535 nm.

### Subcutaneous tumor xenograft assay and metastatic assay

The 6 weeks old female nude mice were purchased from the SLRC Laboratory Animal Center (Shanghai, China) and kept in a specific pathogen free condition. To establish the subcutaneous tumor xenograft model, 6-8 weeks' mice were randomly assigned into two groups (five mice per group), and each nude mouse was injected subcutaneously with 5 × 10^6^ A549 cells (resuspended in 100 μl DMEM medium) transfected with pLenti and pLenti- miR-199a. The diameters of tumor tissue in mice were measured weekly with calipers and the volumes were calculated by the following formula: volume = length × width^2^/2. After the experiment, the mice were sacrificed and the primary tumors were obtained and weighed. To investigate the metastatic ability, mice were injected with 2.5 × 10^6^ cells (resuspended in 200 μl DMEM medium) via the tail vein, and 7 weeks following the injection, the mice were sacrificed and lung tissues were isolated. The removed tumor tissues and lung tissues were subjected to serial sectioning, hematoxylin and eosin staining or immunohistochemistry. All experimental protocols were approved by the Institutional Animal Care and Use Committee of Shanghai University (Shanghai, China).

### Statistical analysis

Experiments were repeated independently at least three times, and the results are expressed as the mean ± S.E.M. Differences between two experimental groups were evaluated by t-test, and considered statistically significant when p<0.05. Graph Pad Prism 5 software was used to analyze the results.

## Results

### MiR-199a-5p is downregulated in tissues from NSCLC patients

**Figure 1:**
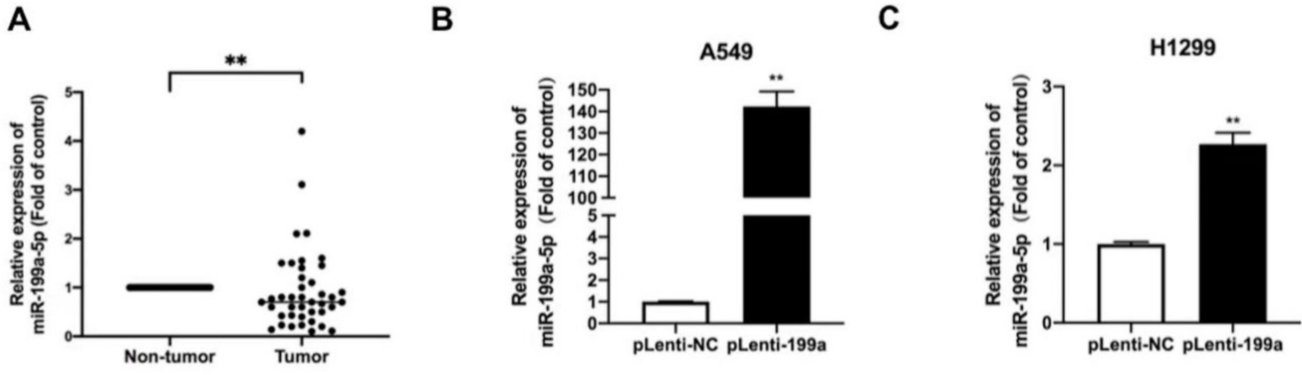
** MiR-199a-5p is low expressed in NSCLC tissues and A stable overexpression of miR-199a-5p.**(A) The expression of miR-199a-5p in cancer and adjacent tissues of NSCLC patients by QRT-PCR. (B&C) The miRNA level of miR-199a-5p was detected highly in two stably overexpressed cell lines. *P < 0.05, ** P < 0.01, *** P < 0.001.

To evaluate the expression of MiR-199a-5p in NSCLC tissues, we performed qRT-PCR on 41 pairs of clinical lung cancer tissues. The result revealed that compared to the miR-199a-5p in non-tumor lung tissues, miR-199a-5p was obviously downregulated in tumor tissues (Fig. 1A).

Plenti-miR-199a H1299 and A549, stable overexpression cell lines containing a plasmid of miR-199a-5p were constructed by qRT-PCR to detect the expression level of miR-199a-5p in the stable transgenic strains of miR-199a in the two cell lines. miR-199a-5p was highly expressed in miR-199a stable cell lines of H1299 and A549 cells (Fig. 1B&C).

### MiR-199a suppresses NSCLC cell growth

**Figure 2:**
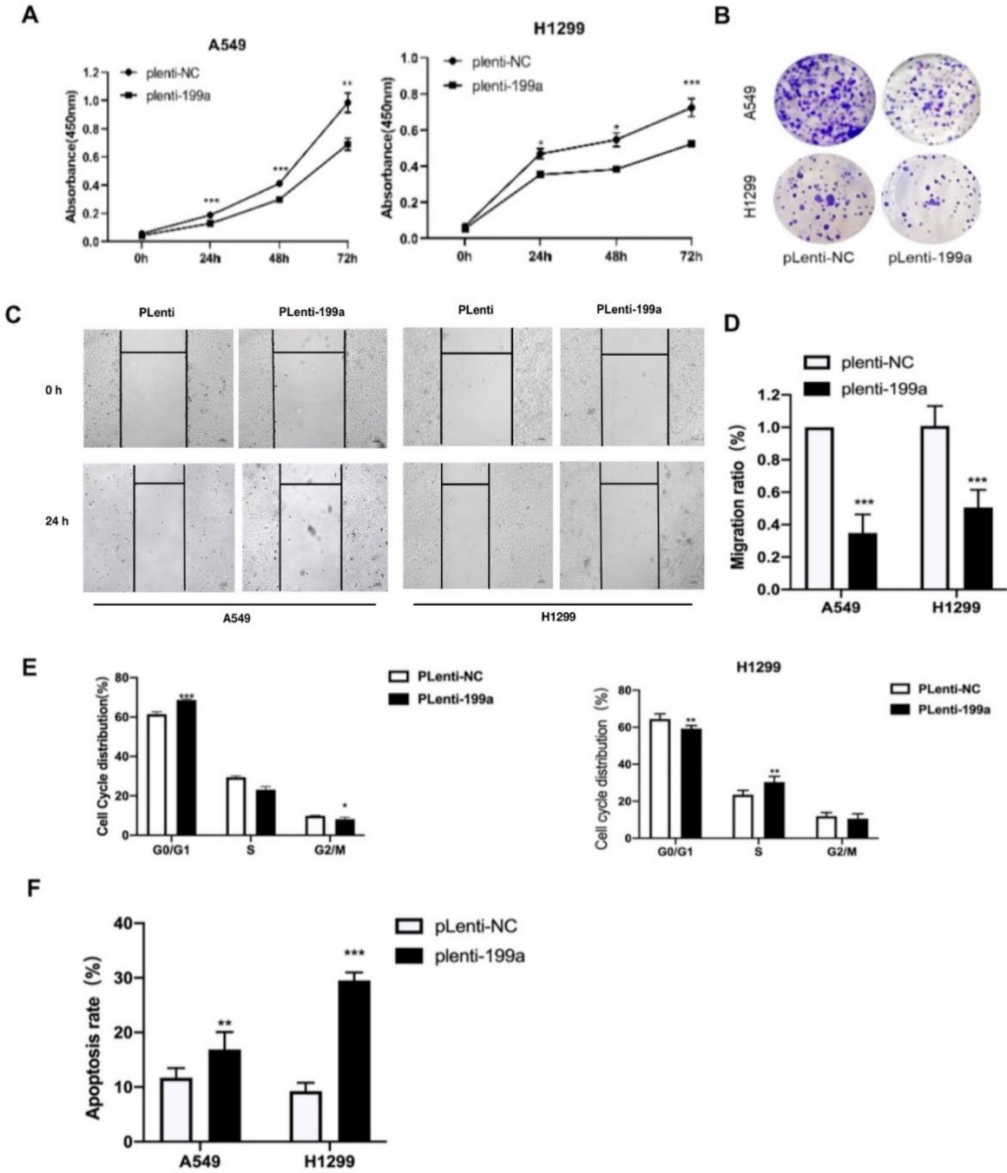
MiR-199a-5p suppresses NSCLC cell proliferation and inhibits both cell migration and cycle, moreover, promotes apoptosis. (A) Using CCK-8 kit to determine the cell proliferation of A549 and H1299. (B) Colony formation emerged in two different transfected cell types in two cell lines. (C&D) Wound healing in A549 and H1299. (E) Flow cytometry tested the cell cycle distribution in A549 and H1299. (F) Flow cytometry analyzed the cell apoptosis distribution in A549 and H1299. *P < 0.05, ** P < 0.01, *** P < 0.001.

We used CCK-8 to find the influence of miR-199a on the proliferation of NSCLC cells and it can be summarized that miR-199a has the ability of inhibiting NSCLC cell proliferation (Fig. 2A). Meanwhile, according to the colony formation assay, miR-199a could effectively inhibit the colony formation rate in two cell lines (Fig. 2B). To sum up, miR-199a is able to suppress the proliferation of NSCLC.

### MiR-199a-5p inhibits NSCLC cell migration and cycle and promotes apoptosis

After being transfected with miR-199a-5p or NC, the migration ratio of NSCLC cells was detected. It revealed that miR-199a-5p could suppress the development of migration in A549 and H1299 (Fig. 2C&D).

In order to study the effect of miR-199a-5p on the cell cycle process of NSCLC, flow cytometry was used to detect the changes in cell cycle distribution with overexpressed miR-199a-5p. The experimental results showed that the stable miR-199a-5p expression can significantly hinder the transition from G0/G1 phase to S phase or G2/M phase (Fig. 2E). Flow cytometry was also used to detect apoptosis distribution with overexpression of miR-199a-5p in NSCLC cells. The conclusion confirmed that the increase of miR-199a-5p level greatly promoted the apoptosis of H1299 and A549 cells (Fig. 2F).

### SLC2A1 is a direct target of miR-199a-5p

**Figure 3:**
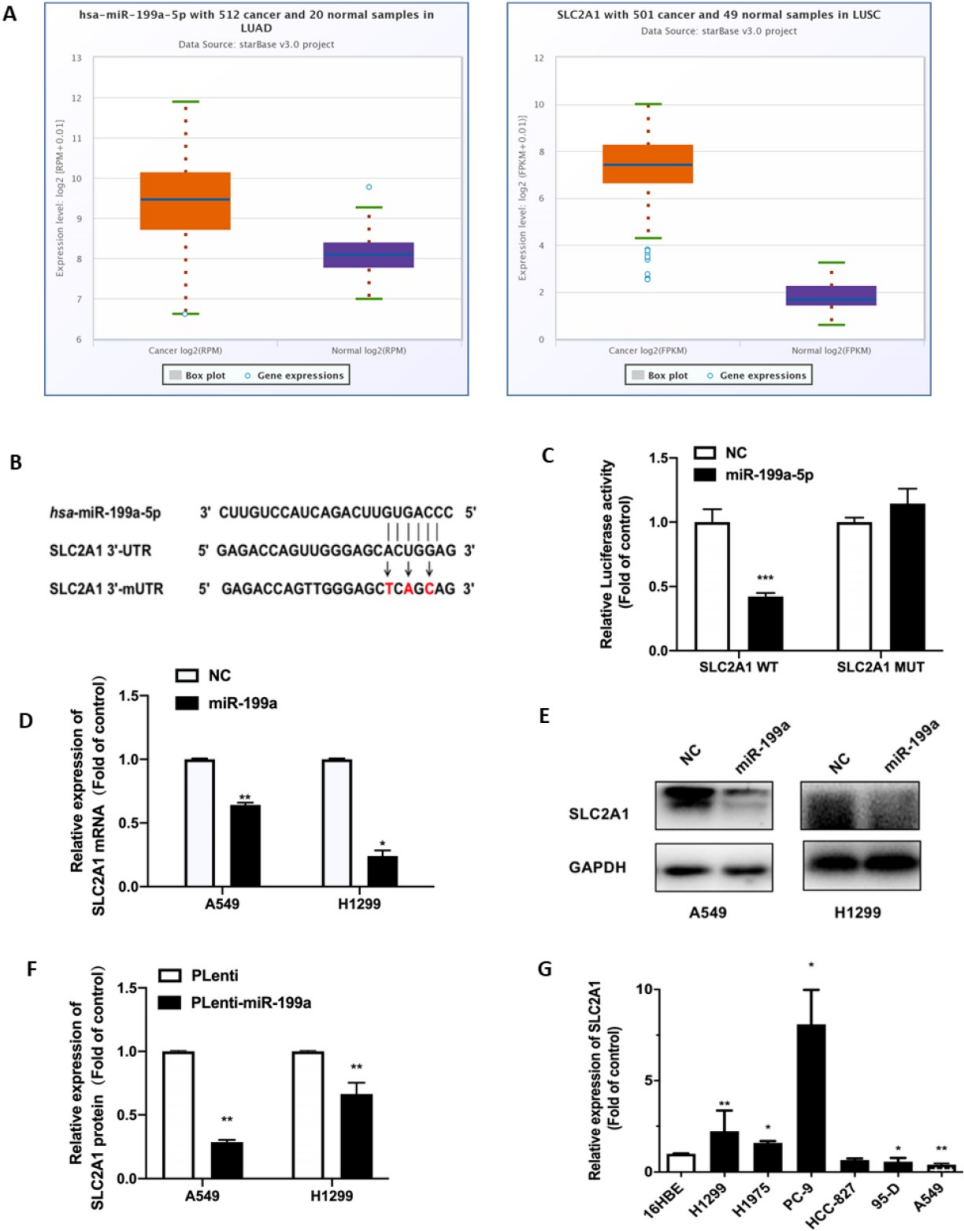
** SLC2A1 is a direct target of miR-199a-5p.** (A) Starbase predict the expression of SLC2A1 in two types of NSCLC (LUAD and LUSC). (B) TargetScan predict the binding site of miR-199a-5p and 3'-UTR of SLC2A1. (C) After the plasma co-infection, the relative activity of the Luciferase was demonstrated by luciferase report. (D-F) miR-199a-5p reduced the mRNA and protein expression of SLCA1. (G) The mRNA level of SLC2A1 in different cell lines of lung were measured. *P < 0.05, ** P < 0.01, *** P < 0.001.

The high expression of SLC2A1 in NSCLC was found through the starbase website (Fig. 3A). SLC2A1 was also found to be a potential target with binding sites for miR-199a-5p by analyses of bioinformatics prediction sites by TargetScan (http://www.targetscan.org/vert_72/). Therefore, SLC2A1 3'UTR containing the targeted binding of miR-199a-5p was inserted into firefly luciferase expression vector pGL3-mireport to construct the wild-type recombinant plasmid, which was denoted as pGL3-SLC2A1-WT-3'UTR. At the same time, interval mutation was performed on the binding sites of miR-199a-5p and SLC2A1 3'UTR to construct the corresponding mutant recombinant plasmid, which was identified as pGL3-SLC2A1-MUT-3 'UTR (Fig. 3B).

In order to verify that SLC2A1 is the direct target gene of miR-199a-5p, all plasmids were co-transfected into HEK-293T cells and verified by changes in luciferin activity. The experimental results showed that luciferin activity was decreased in the wild-type SLC2A1 3'UTR with overexpression of miR-199a, while it did not significantly change in the mutant SLC2A1 3'UTR with overexpression of miR-199a (Fig. 3C).

At the same time, this study found that miR-199a could effectively reduce both SLC2A1 mRNA and protein levels in H1299 and A549 stable transgenic cells of Plenti/Plenti-miR-199a (Fig. 3D-F). We used qRT-PCR to determine the mRNA level of SLC2A1 in NSCLC cell lines and it verified that the level of SLC2A1 was considered to be much higher in lung cancer cell lines than that in normal epithelial cell lines (Fig. 3G).

### Downregulation of SLC2A1 suppresses NSCLC cell proliferation

**Figure 4:**
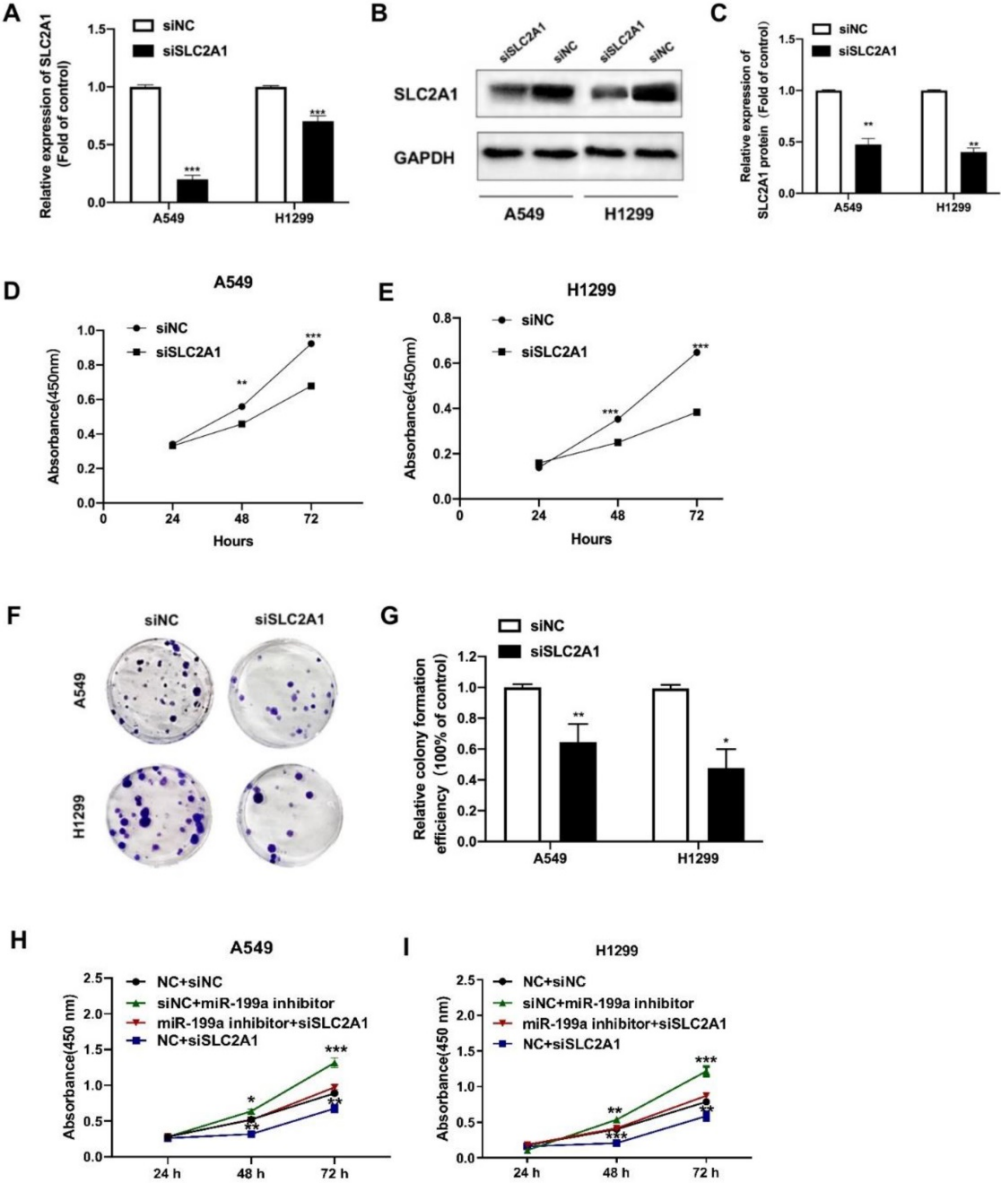
** Silencing SLC2A1 suppresses NSCLC cell proliferation.** (A) The transfection efficiency of SLC2A1 in H1299 and A549 cells was detected by qRT-PCR. (B-C) Western blot and its analysis of SLC2A1 protein level in NSCLC cell lines transfected with siSLC2A1 or siNC. (D-E) A CCK-8 kit was taken to determine the cell proliferation of NSCLC cell lines transfected with siSLC2A1 or siNC. (F-G) Colony formation assay of NSCLC cell lines transfected with siSLC2A1 or siNC. (H-I) CCK-8 assay was used to detect the cell proliferation after co-transfection by rescue test. *P < 0.05, ** P < 0.01, *** P < 0.001.

To further study the oncogenic effect of SLC2A1, siRNA was inserted to knock down SLC2A1, and then its effect was explored on cell phenotype. After transfected with siSLC2A1, both mRNA and protein level of SLC2A1 evidently declined in NSCLC cell lines (Fig. 4A-C). We used CCK-8 to show that the proliferation of NSCLC cell lines was suppressed by siSLC2A1 (Fig. 4D-E). Colony formation assay was also performed to find the results of silence of SLC2A1 and the number of colonies in A549 and H1299 decreased (Fig. 4F-G). These consequences indicated that the proliferation of NSCLC cells could be suppressed by the downregulation of SLC2A1.

Besides, we carried out the rescue experiment by transfecting siSLC2A1 and miR-199a inhibitor to explore whether the SLC2A1 can reverse the effect of miR-199a. The results revealed that knockdown of SLC2A1 expression could reverse the effect of miR-199a on the proliferation of NSCLC cells and verify miRNA-199a-5p exerting its function in NSCLC via regulating SLC2A1 (Fig. 4H-I).

### Knockdown of SLC2A1 suppresses NSCLC cell glycolysis

**Figure 5:**
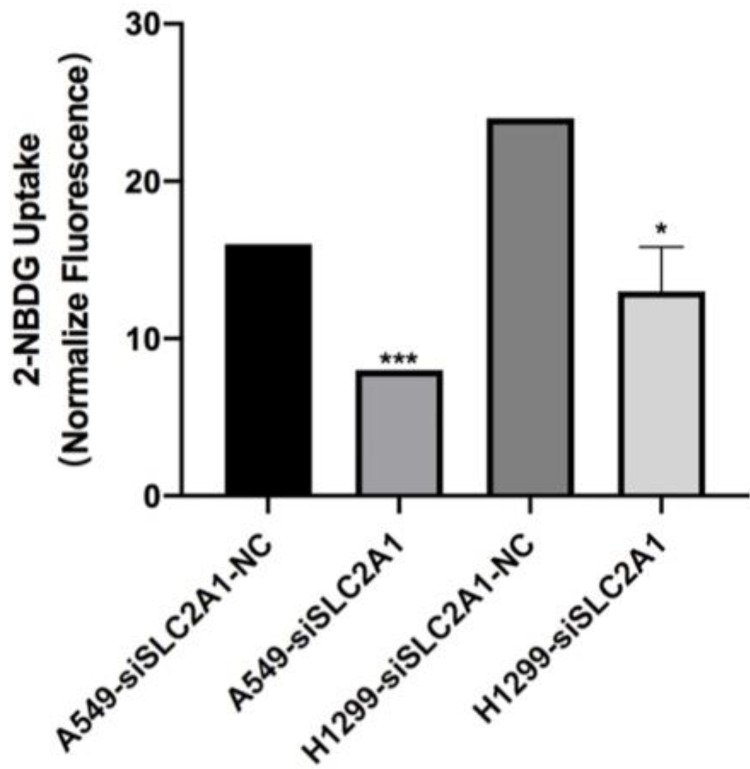
Fluorescence is measured after 2-NBDG incubated with SLC2A1 or NC siRNA. *P < 0.05, ** P < 0.01, *** P < 0.001.

2-NBDG was considered as a fluorescent indicator for direct glucose uptake measurements. Results proved that knockdown of SLC2A1 could suppress glycolysis in A549 and H1299 (Fig. 5).

### Knockdown of SLC2A1 reduces cell migration, arrests cell cycle and promotes apoptosis in NSCLC

**Figure 6:**
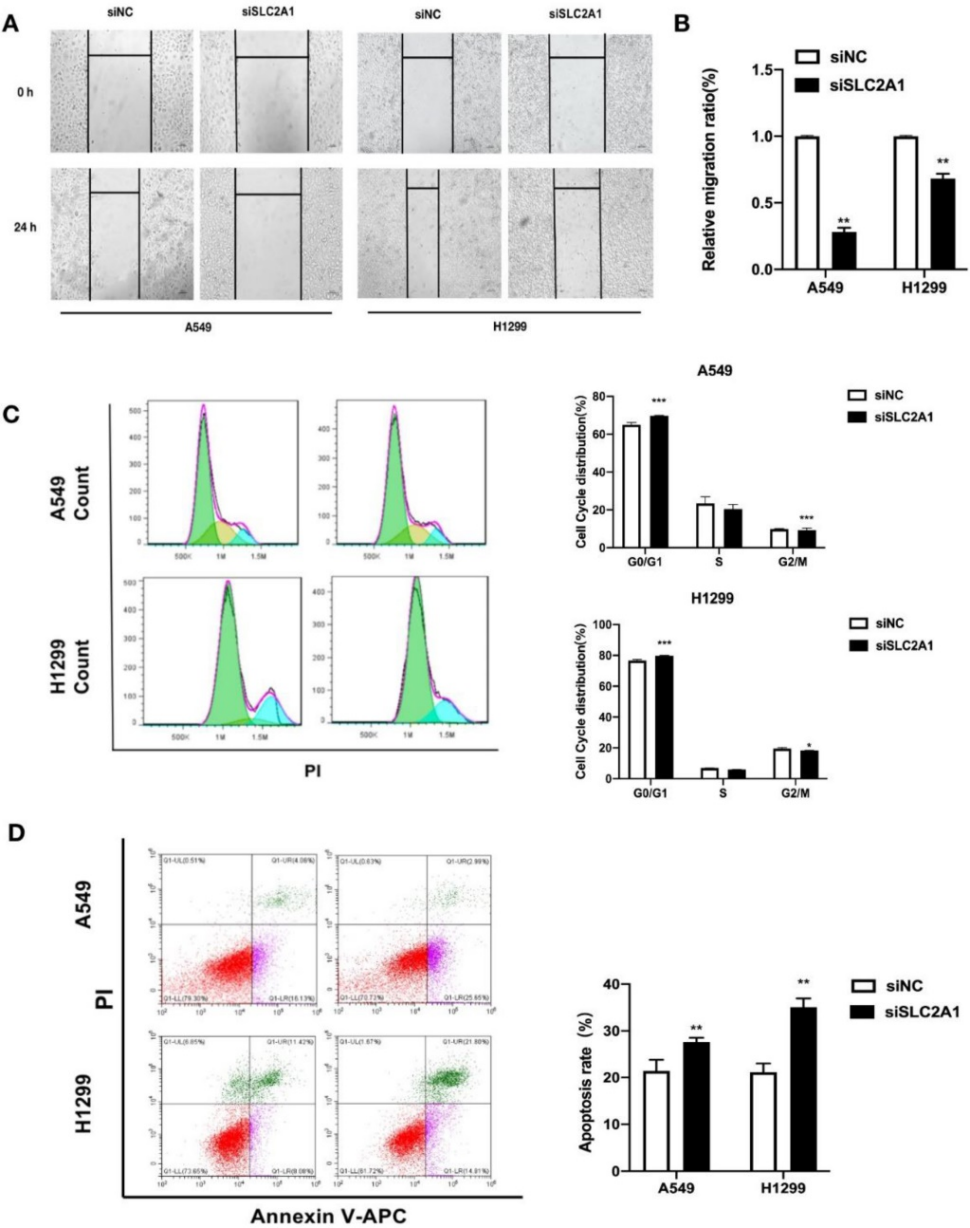
** Downregulation of SLC2A1 reduces both cell migration and cell cycle and promotes apoptosis.** (A&B) The speed of migration was detected by taking a wound healing in two cell lines. (C) Flow cytometry made the analysis of cell cycle after NSCLC cell lines transfected with siSLC2A1 or siNC. (D) Flow cytometry analyze the distribution of cell apoptosis in NSCLC cell lines after transfected with siSLC2A1 or siNC. *P < 0.05, ** P < 0.01, *** P < 0.001.

We took wound healing assay after silencing SLC2A1 to indicate the ability of SLC2A1 in cell migration. H1299 and A549 cells transfected with siSLC2A1 migrated slower than the group of siNC. It is suggested that downregulating SLC2A1 could control NSCLC cell migration (Fig. 6A&B).

For the purpose of investigating the distribution of cell cycle and changes of cell apoptosis after SLC2A1 knockdown, the cell cycle and apoptosis were measured by flow cytometry. The results confirmed that down-regulation of SLC2A1 could effectively block the periodic transformation of H1299 and A549 (Fig. 6C) and promote the apoptosis of cancer cells (Fig. 6D).

### miR-199a suppresses tumor growth in A549 xenograft mice via targeting *SLC2A1*

**Figure 7:**
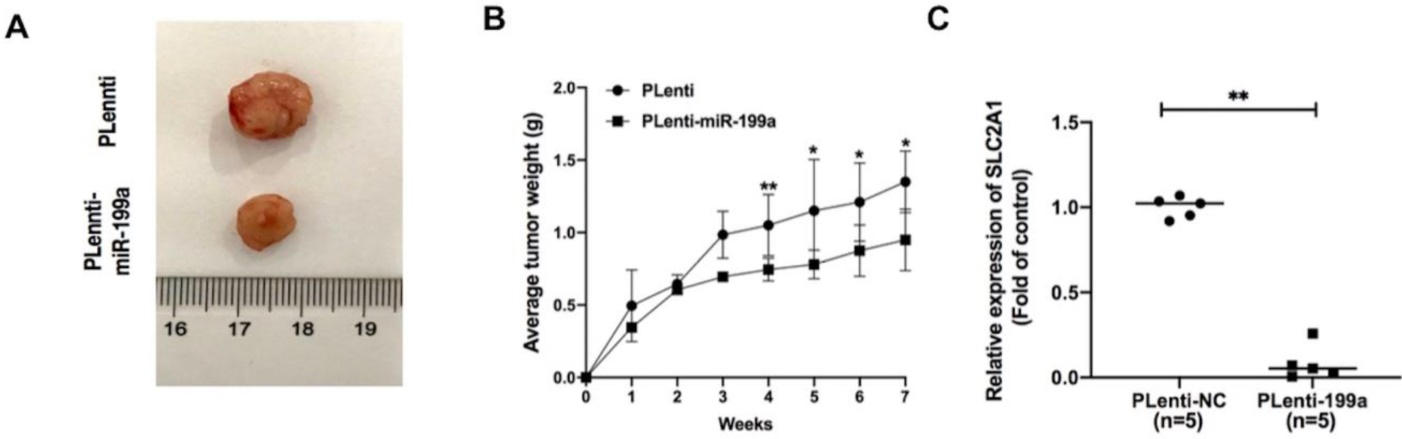
** miR-199a inhibits the development of tumor.** (A) plenti-NC tumor samples were bigger than Plenti-miR-199a tumor samples. (B) plenti-NC tumor samples were heavier than that of plenti-miR-199a tumor samples. (C) mRNA expression of SLC2A1 was tested in plenti/Plenti-199a animal models by qRT-PCR. *P < 0.05, ** P < 0.01, *** P < 0.001.

In A549 xenograft mice, the tumor samples from plenti-miR-199a group were significantly smaller than that of control group (Fig. 7A) and the weight of plenti-miR-199a tumor samples was continuously smaller than that of plenti group during the 7 weeks (Fig. 7B). As miR-199a was proved effectively inhibit the development of tumor, the mRNA expression of SLC2A1 was tested in the tumor samples from plenti/plenti-miR-199a mouse models which were five in number respectively. It suggested that the mRNA expression of SLC2A1 was remarkably downregulated in the tumors of the plenti-199a lung cancer mice compared with that of the plenti-NC lung cancer mice (Fig. 7C).

## Discussion

This research studied the effects of miR-199a-5p and its target gene SLC2A1 on cell proliferation, migration, cell cycle and the apoptosis of NSCLC, providing new molecular targets and theoretical foundation for the early diagnoses and treatments of NSCLC.

SLC2A1 was predicted and verified as the direct target gene of miR-199a-5p through a variety of target gene prediction websites and gene experiments reported by dual luciferase. In addition, according to the database, SLC2A1 is significantly highly expressed in tissue samples and cell lines of NSCLC patients, and the high expression of SLC2A1 is negatively correlated with the overall survival rate of lung cancer patients. This suggests that SLC2A1 may play a role as an anti-oncogene in NSCLC. Subsequently, H1299 and A549 cell phenotypes were detected by using siRNA (Small RNA, siRNA) which knocked down SLC2A1 expression. The phenotypic results confirmed that SLC2A1 was a suppressor gene.

In conclusion, miR-199a-5p acts as a suppressor gene of NSCLC and the regulatory mechanism between miR-199a-5p and the target gene SLC2A1 may provide new targets and strategies for the early diagnoses and precise treatments of NSCLC.

Gluts play a key role in the treatment of diseases, for example, it can effectively inhibit the occurrence of various types of tumors and promote the wound healing caused by diabetes. SLC2A1, a glucose-promoting transporter, is expressed in most tissues of human bodies. It is considered to be a major member of the GLUT family that regulates the basic transport of hexose carbohydrates in many different types of cell as its 1-2 mmol/L K_m_ for glucose. SLC2A1 is expressed at the highest levels in red blood cells, neuronal membranes, blood-brain barrier, eyes, placenta, and lactating mammary glands. SLC2A1 is also involved in the metabolism of hepatocytes, including hepatocytes and non-parenchymal cells^9^. SLC2A1 is a transporter that mainly mediates the regulation of tumor energy metabolism. The high expression of SLC2A1 accelerates the glucose uptake of tumor cells and provides sufficient raw materials for glycolysis. While providing energy for tumor, fatty acids and nucleic acids produced in the process also promote tumor growth and metastasis^10^. SLC2A1, which encodes SLC2A1 protein, was found highly expressed in hepatocellular carcinoma (HCC) and gastric cancer (GC) tissues and was proved that deregulating it could promote tumor cell proliferation and metastasis^11^, ^12^. Concerning lung cancer, SLC2A1 was observed highly expressed in patients of Lung adenocarcinoma (LUAD) which its development could be inhibited by SLC2A1 through various molecular pathways^13^. Ding et al. found that GW501516, an agonist of peroxisome proliferators activated receptors (PPARs), significantly increased the expression of SLC2A1 in HeLa cells, SW480, HCT-116 and other cancer cell lines^14^. Many studies have pointed out that SLC2A1 plays a key role in mediating the regulation of tumor energy metabolism, indicating that it is a pivotal potential target in tumor therapy.

The change of cell metabolism is a basic adaptation of growth factor over stimulation in the process of rapid tumor proliferation^15^. In recent years, the development of the studies of glucose metabolism of tumor provides a new strategy for the treatment of a variety of malignant tumors, and also shows the importance of precision medicine^16^. Compared with normal tissues, the glucose metabolism of tumor cells was enhanced. The resulting increase in glucose demand requires a corresponding increase in glucose transport across the plasma membrane^17^-^19^. Glut family members are overexpressed in most cancers. Because unlimited proliferation requires energy, cancer cells usually express glut which is normally high presented in normal tissues^20^. Overexpression of SLC2A1 is associated with many tumor features, including increased invasion, increased proliferative activity, and decreased survival. The expression of SLC2A1 has been proved to be related to the hypoxia level of tumor. Hypoxia can increase SLC2A1 gene expression^21^, ^22^. Due to the complex mechanism of tumor glycolysis and the lack of selective, effective and safe glycolysis inhibitors, the mechanism of action of GLUT protein family in malignant tumors is still unclear, and its targeted therapy has not been successful. However, more and more subtypes of GLUT protein family have been confirmed as a new class of anti-tumor targets. At the same time, GLUT plays an important regulatory role in diabetes, heart disease and tissue diseases associated with GLUT protein spectrum^23^-^25^.

MiR-199a-5p is down regulated in a variety of malignant tumors, and its low expression is associated with poor prognoses. The methylation level of miR-199a promoter in NSCLC tissues was significantly higher than that in adjacent tissues, and overexpressing of miR199a-5p could inhibit the proliferation and tumorigenicity of NSCLC cells^26^. It has been proved that AKAP1, SNAI1 and Caprin1 could be target genes of miR-199a-5p, and their overexpression worsens tumor developments^27^-^29^. At the same time, miR-199a-5p can form pvt1 / miR-199a / caveolin1 signaling pathway for lung cancer cells that are fully exposed to PM2.5, which can affect the anti-tumor activity of lentinan^30^. MiR-199a-5p acts as a tumor suppressor in other kinds of tumors as well. For example, in papillary thyroid carcinoma, it can target NNT-AS1 to inhibit the proliferation, migration and invasion of PTC cells^31^.

Non-coding RNA targeting glucose transporters can regulate tumorigenesis and disease progression more precisely. Therefore, the use of miRNA mimics and inhibitors in clinical can make delivery to target organs more accurate and effective, which creates an attractive therapeutic prospect^32^. However, our overall control of miRNA regulatory network is not comprehensive enough. For example, a metabolic enzyme in the metabolic pathway is often regulated by multiple miRNAs. As miRNA may have multiple targets and functional interactions between miRNAs occur, it is worthy of further experimental verification^33^, ^34^. Therefore, it is necessary to determine the complete expression and targeting effect of miRNA before clinical application. The regulation of GLUT family by non-coding RNA is still in the exploratory stage. For example, the encoding mechanisms of other non-coding RNA, tRF, circRNA and their interactions achieve a very worthwhile exploring and studying.

In conclusion, glucose transporter glut is an effective target for cancers and a variety of diseases, and miR-199a-5p works efficiently in inhibiting the occurrence and development of variety kinds of tumors. Additionally, non-coding RNA can be more precisely regulated, which provides a new approach to clinical diagnoses and treatments of diseases.

## Supplementary Material

Supplementary tables.

## Abbreviations

NSCLC: non-small cell lung cancer
miRNAs: MicroRNAs
SLC2A1: solute carrier family 2 member 1
GLUT1: glucose transporter 1
HCC: hepatocellular carcinoma
GLUTs: glucose transporter family
CCK-8: Cell Counting Kit-8
qRT-PCR: Quantitative real-time PCR
GC: gastric cancer
LUAD: Lung adenocarcinoma
PPARs: peroxisome proliferators activated receptors
