# Supplementary material for: miRNA-199a-5p/SLC2A1 axis regulates glucose metabolism in non-small cell lung cancer
Source: J Cancer. 2022 Apr 18;13(7):2352–61. doi: 10.7150/jca.67990 (PMC9066207; doi:10.7150/jca.67990)
Supplement: Supplementary file 1 — Supplementary tables. [file jcav13p2352s1.pdf]

Table 1: Details of 41 NSCLC patients donating tissue samples

No.	Age	Gender	Histologic Type	pTNM
1	54	F	Adenocarcinoma	T2N2M0
2	64	M	Squamous cell carcinoma	T2N1M0
3	49	M	Adenocarcinoma	T2N0M0
4	73	M	Adenocarcinoma	/
5	60	M	Adenocarcinoma	T4N0M0
6	72	M	Adenocarcinoma	T2N2M0
7	40	M	Adenocarcinoma	T1N1M0
8	66	M	Adenocarcinoma	T2N2M0
9	67	M	Adenocarcinoma	T2N0M0
10	67	F	Adenocarcinoma	T2N0M0
11	76	M	Squamous cell carcinoma	T2N0M0
12	80	F	Adenocarcinoma	T1N0M0
13	40	F	Adenocarcinoma	T2N2M0
14	76	F	Adenocarcinoma	T1N0M0
15	72	M	Large-Cell Neuroendocrine Carcinoma	T2N2M0
16	67	M	Squamous cell carcinoma	T2N2M0
17	58	M	Adenocarcinoma	T2N0M0
18	49	M	Adenocarcinoma	T2N0M0
19	54	M	Squamous cell carcinoma	T1N0M0
20	60	F	Adenocarcinoma	T2N0M0
21	77	F	Squamous cell carcinoma	T2N1M0
22	51	M	Squamous cell carcinoma	T1N1M0

23	75	M	NSCLC	T1N2M0
24	66	M	Adenocarcinoma	T1N0M1
25	63	M	Squamous cell carcinoma	T2N1M0
26	59	M	Squamous cell carcinoma	T2N2M0
27	75	M	Adenocarcinoma	T1N0M0
28	47	F	Adenocarcinoma	T1N2M0
29	64	M	Adenocarcinoma	T2N0M0
30	66	M	Adenocarcinoma	T2N0M0
31	68	M	Adenocarcinoma	T2N0M0
32	58	M	Adenocarcinoma	T2N0M0
33	78	M	Adenocarcinoma	T2N0M0
34	74	F	NSCLC	T2N2M0
35	65	M	Adenocarcinoma	T2N0M0
36	56	F	Adenocarcinoma	T1N2M0
37	59	M	NSCLC	T2N2M0
38	38	M	Adenocarcinoma	T2N0M0
39	44	F	Adenocarcinoma	T2N1M0
40	68	F	Adenocarcinoma	T2N0M0
41	74	M	Squamous cell carcinoma	T2N1M0

Table 2: Details of primer sequences

Primer Name	Primer Sequence (5'-3')
U6 snRNA(F)	CTCGCTTCGGCAGCACA
U6 snRNA(R)	AACGCTTCACGAATTTGCGT
18S RNA(F)	CTCGTTCGGCAGCACA
18S RNA(R)	GCCTCACTAAACCATCCAA
miR-199a-5p qRT	CCCAGTGTTCAGACTACCTGTTC
SLC2A1 3'-UTR(F)	GGGAGCTCAGCAGTGCAGGGAGGAGAGG
SLC2A1 3'-UTR(R)	GCACTGCTGAGCTCCCAACTGGTCTCAG
SLC2A1 qRT(F)	CAGATGATGCGGGAGAAGAA
SLC2A1 qRT(R)	CCAAAGCGGTTAACGAAAAGG
